# Wound healing and antibacterial properties of methanolic extract of *Pupalia lappacea* Juss in rats

**DOI:** 10.1186/1472-6882-14-157

**Published:** 2014-05-13

**Authors:** Sunday Ositadinma Udegbunam, Rita Ijoema Udegbunam, Chijioke Clement Muogbo, Madubuike Umunna Anyanwu, Chinaka Onyebuchi Nwaehujor

**Affiliations:** 1 Department of Veterinary Surgery, Faculty of Veterinary Medicine, University of Nigeria, Nsukka, Nigeria; 2 Department of Pharmaceutics, Faculty of Pharmaceutical Sciences, University of Nigeria, Nsukka, Nigeria; 3 Department of Veterinary Pathology and Microbiology, Faculty of Veterinary Medicine, University of Nigeria, Nsukka, Nigeria; 4 Department of Biochemistry, Faculty of Basic Medical Sciences, University of Calabar, Calabar, Nigeria

**Keywords:** *Pupalia lappacea*, Wounds, Antibacterial, Ointments

## Abstract

**Background:**

Wound healing is a natural process that enables tissue repair after an injury. To shorten its duration and minimize associated complications, wounds are treated with medications. Currently there is a growing interest in the use of alternative wound dressing agents such as plant extracts. One plant used traditionally in wound treatment is *Pupalia lappacea*. In view of its use in wound care, we investigated the wound healing activities of 80% methanolic leave extract of *Pupalia lappacea* using excision, incision and dead space wound models. Also its effects on three common wound contaminants were investigated.

**Methods:**

Excision wounds were created, contaminated with microbes and treated with ointments (10% and 20% *w/w*) prepared from *Pupalia lappacea.* Incision and dead space wounds were also created in rats which were subsequently dosed orally with the extract. The wound healing activities of *Pupalia lappacea* ointment on excision wound was assessed by rates of wound contraction and epithelialization as well as its antibacterial effects. The effects of *Pupalia lappacea* on incision and dead-space wounds were determined by the wound breaking strengths and weights of the granuloma tissues formed, respectively.

**Results:**

*Pupalia. lappacea* ointments significantly (p < 0.05) accelerated wound healing with 20% ointment having the highest percentage wound contraction and rate of epithelialization. At 4, 7 and 14 days post treatment, mean total viable bacterial count of excision wounds of the extract treated groups were significantly (p < 0.05) lower compared against the control. Wound breaking strengths and weights of granuloma tissues formed in the extract treated groups were significantly (p < 0.05) higher than those of the control group. The minimum inhibitory concentration values obtained for the *Pupalia lappacea* extract against *Pseudomonas aeruginosa*, *Staphylococcus aureus* and *Bacillus subtilis* were 9 mg/ml, 4 mg/ml and 3 mg/ml, respectively, while the corresponding minimum bactericidal concentrations were 10 mg/ml, 8 mg/ml and 7 mg/ml.

**Conclusion:**

The results obtained showed that *Pupalia. lappacea* has good wound healing and antibacterial activities. These findings validate the use of this plant in traditional medicine for treatment of wounds.

## Background

A wound refers to an injury to the skin or underlying tissues or organs [1]. Dermal wounds are often caused by surgery, trauma, and chemicals or as a result of diseases [1,2]. Intentionally created dermal wounds can be incisional, whereby the wound is brought about by surgical cutting into the skin with a scalpel or excision wound created when a part of the skin is cut off [3]. The process of tissue repair after an insult to the tissue (wound) is called ‘wound healing’ [4]. Wound healing is an intricate process in which usually the skin repairs itself [5]. The process involves four overlapping phases: haemostasis (ceasation of bleeding), inflammation, proliferation, and remodeling [4,5].

In haemostasis, thromboxane synthase (TXS) converts prostaglandin H2 into thromboxane A2 which causes vasoconstriction and platelet aggregation [6]. Failure of the haemostatic process is prevented by plasminogen activator inhibitor type 1 (PAI-1), an inhibitor of fibrinolysis [7]. In the inflammation phase, neutrophils release free radicals to kill wound contaminating bacteria [8,9]. Heme and heme proteins which accumulate at the local site of the wound have prooxidative and proinflammatory properties by inducing the expression of adhesion molecules, causing vascular permeability and leukocyte infiltration. These actions initiate wound healing process [5]. Conversely, heme oxygenase-1 (HO-1) has anti-inflammatory and antioxidant activities and is responsible for a wide range of wound healing functions. It converts heme into biliverdin/bilirubin, iron and carbon monoxide, which are potent antioxidant products [5]. In proliferation phase, overexpression of HO-1 helps to accelerate wound healing such as amelioration of inflammation, proliferation and protection against endothelial cell apoptosis [10]. In remodeling phase, matrix metalloproteinases (MMPs) plays a major role in wound healing by extracellular matrix (ECM) remodeling [11].

The length of time it takes for wound healing process to be complete is dependent on the rate of availability of biochemical substances required for each phase to occur. To shorten the duration of the healing process and minimize complications (such as overwhelming bacterial wound contamination) of natural healing, medical professionals use orthodox medications (which increase the rate of healing process and/or control secondary microbial wound contamination) to treat wounds [12]. But, the prices of these orthodox medications are exorbitant and many wound contaminating bacteria are becoming increasingly resistant to their effects [13].

In traditional medicine, medicinal plants are used in the preparation of decoctions which are applied to dermal wounds to enable healing [14]. The efficacies of these plants in wound healing have been experienced and passed on from one generation to the other. One of these medicinal plants widely used traditionally in Nsukka Southeast, Nigeria in treating wounds is *Pupalia lappacea* (Family Amaranthaceae). The common name is forest Burr or creeping cork’s comb. It is an erect or straggling under shrub with flowers in spicate clusters, and it usually grows along hedges [13]. It is used in agrihorticulture as a fodder [15]. Traditional practitioners including those in Nsukka, claim that foliage parts of *P. lappacea* are used in form of poultices and decoctions to treat different ailments such as bone fractures and inflammatory conditions, boils, cough, fever, leprosy sores, flatulence, jaundice, abdominal colic, cephalgia, diarrhoea, paralysis, erectile dysfunction, vomiting, toothache, cuts, chronic wounds, fever and malaria [13,16-20]. For the treatment of wounds, the leaf paste and the fruit juice is applied externally [13]. Scientific investigations have validated some of these claims such as antimalaria [20], anticancer [13], antinociceptive and antipyretic [18] activities of the *P. lappacea* extract. No study has been done to evaluate its wound healing and antibacterial activities.

Moreover, chemical investigations of *P. lappacea* leaf extract revealed that it consists of 8 compounds which included: 1-docosanol, stearic acid, stigmasterol, sitosterol, N-benzoyl-L-phenyl alaninol acetate, setosterol-3-O-D-glucopyranoside, stigmasterol-3-O-D-glucopyranoside and 20-hydroxyl ecdysone [21]. These biochemical compounds have been shown to promote wound healing process in animals and humans. Stigmaesterol have been shown to exhibit haemostatic [22] and anti-inflammatory activities [5,23]. The 20-hydroxyl ecdysyne promoted protein synthesis and wound healing in animals and humans [24]. Docosanol was used by to treat herpes simplex virus cutaneous wounds in mice [25]. Plant extract that contains N-benzoyl-L-phenyl alaninol exhibited antibacterial activity [26]. Since some of the compounds contained in *P. lappacea* leaf extract showed activities involved in wound healing process, the plant extract may exhibit wound healing activities in animals and humans as claimed by the traditional practitioners. The objective of this study was therefore to evaluate the wound healing and antibacterial activities of *P. lappacea* methanolic leaf extract in rats.

## Methods

The experimental protocols used in this study was approved by the Ethics Committee of the University of Nigeria, Nsukka and conforms with the guide to the care and use of animals in research and teaching of University of Nigeria, Nsukka, Enugu State Nigeria.

### Animals

A total of 92 8-week-old male albino *Wistar* rats weighing between 129 and 131 g were obtained from the laboratory animal unit, Faculty of Veterinary Medicine, University of Nigeria, Nsukka. They were fed on commercial growers mash (Top feeds®) and water was provided *ad libitum*. These rats were acclimatized for 2 weeks in the animal house at the Department of Veterinary Surgery, University of Nigeria, Nsukka.

### Plant collection and identification

Fresh leaves of *Pupalia lappacea* were collected from Obollo-Afor in Nsukka Local Government Area Enugu State, Nigeria, in the month of March 2013 and were identified at the International Center for Ethnomedicine and Drug Development (InterCEDD), Nsukka, by a plant taxonomist, Mr. A. Ozioko. Samples were registered and deposited in the center’s herbarium.

### Extraction

Half a kilogram of the leaves were air dried at room temperature for 2 weeks and then pulverized using the laboratory grinding machine at the Department of Crop Science, University of Nigeria, Nsukka. The pulverized leaves were macerated in 80% methanol for 48 hr with intermittent vigorous shaking at every 2 hr. After 48 hr, the mixture was filtered and the extract concentrated using a rotary evaporator set at 40°C. The dried extract was weighed and the percentage yield calculated. The extract was then stored at 4°C in a refrigerator before use.

### Acute toxicity test

Thirty adult rats were randomly divided into six groups of five animals per group. The animals were deprived water for 16 hr before administration of the extract. The increasing doses of the extract 200, 400, 600, 1000 and 2000 mg/kg body weight suspended in dimethylsulfoxide (DMSO) was administered orally to the test groups, respectively, using a ball-tipped intubation needle fitted onto a syringe. The last group received 1 ml/kg of DMSO and served as the control. The rats were allowed access to food and water *ad libitum* and were observed for 48 hr for behavioural changes and death. The time of onset, intensity, and duration of these symptoms, if any, was recorded.

### Phytochemical analysis for bioactive substances

The methanolic leaf extract was screened for the presence of bioactive components following the methods of Trease and Evans [27].

### Preparation of ointments

The method of Okore *et al*. [28] was adopted in preparation of two herbal ointments containing 10% w/w and 20% w/w of the extract in sterile soft white paraffin. Immediately after preparation, the ointments were aseptically transferred into sterile cream tubes and sealed.

### Pathogens and preparation of inocula

The bacterial organisms - *Pseudomonas aeruginosa*, *Stapylococcus aureus* and *Bacillus subtilis* used in this study were collected from the Department of Pharmaceutics, University of Nigeria, Nsukka. They were clinical wound isolates from patients in Nsukka, Nigeria, fully identified and maintained on nutrient agar slope at 4°C at the Department of Pharmaceutical Microbiology Laboratory, University of Nigeria, Nsukka. Prior to use, the organisms were sub-cultured on sterile nutrient agar plate, incubated aerobically at 37°C for 24 hr. Colonies of each organism was homogenized in sterile phosphate buffered saline (PBS) and the turbidity adjusted to correspond to 0.5 McFarland’s turbidity standard (equivalent to 1 × 10^8^ cfu/ml). The standardized broth cultures were kept at 4°C until needed.

### Determination of minimum inhibitory concentration (MIC) and minimum bactericidal concentration (MBC)

MIC of the extract was determined by macro broth dilution following the method described by Baron and Finegold [29]. Dilution of *P. lappacea* extract was made to various concentrations of 1, 2, 3, 4, 5, 6, 7, 8, 9 and 10 mg/ml in series of test tubes. Equal volume of the extract and sterile nutrient broth were mixed. Specifically, 0.1 ml of standardized inoculum (1 × 10^8^ cfu/ml) of each of the test organism was added to each tube. The tubes were incubated aerobically at 37°C for 24 h. Two control tubes were maintained for each test batch. These included antibiotic control (tube containing extract and the growth medium without inoculum) and organism control (the tube containing the growth medium, physiological saline and the inoculum). The lowest concentration (highest dilution) of the extract that produced no visible bacterial growth (no turbidity) when compared with the control tubes were regarded as MIC. However, the MBC was determined by sub-culturing the test dilution on to a fresh drug-free nutrient agar and incubated further for 24 hr. The highest dilution that yielded no single bacterial colony on a solid medium was taken as MBC.

### Wound healing studies

#### Creation and contamination of excision wound

Thirty two rats were anaesthesized with 10 mg/kg body weight of xylazine hydrochloride and 50 mg/kg ketamine hydrochloride. Their dorsum was shaved and disinfected with methylated spirit. Then, full thickness excision wounds were created following the method described by Morton and Malone [30].

Post wounding, the rats were randomly assigned into 4 groups of 8 animals per group. Then, using sterile Pasteur pipettes, wound on each animal was contaminated by flooding with 1 ml of standardized broth culture of each test organism. To minimize further microbial contamination of wound, each animal was carefully placed individually in disinfected cages kept in a disinfected, clean and dust-free animal house in the Department of Veterinary Surgery, University of Nigeria, Nsukka. The wounds were not treated for 24 hr post contamination to ensure colonization and establishment of infection. Treatment of animal wound commenced 48 hr post contamination. Four treatment groups consisting of 5 animals per group were treated as follows: groups I and II were treated topically with 20% and 10% *P. lappacea* leaf extract ointment (PLLEO), respectively, while groups III and IV were treated with gentamicin sulphate and sterile soft white paraffin, respectively. Treatment of the animals continued until complete healing occurred.

#### Percentage wound contraction

The wound diameter of each animal was measured at 7, 10, 13, 16, 19, 21, 24 and 27 days post wounding (dpw) using a transparent meter rule, and the percentage wound contraction calculated using the method of Ezike *et al*. [31].

#### Wound epithelialization

Period of wound epithelialization was calculated as the number of days required for the scar to fall off leaving no raw wound [32]. The mean days for wound epithelialization for each group were calculated.

#### Determination of wound microbial load

At 4, 7 and 14 days post-treatment (dpt), wound swabs from each animal was taken in duplicate using sterile swab sticks. The total viable count of the wound was determined following the standard plate counting method [33]. Briefly, each swab was inoculated into sterile nutrient broth to make stock solutions which was used for double-fold (10^-2^) dilutions. Using a sterile pipette, 0.1 ml of the 10^-2^ dilution was introduced on the surface of sterile nutrient agar and a sterile glass spreader was used for even distribution of the inoculum. The inoculated plates were incubated at 37°C for 24 hours and the colonies were counted using a colony counter.

#### Incision wound

The method of Rathi *et al*. [34] was adopted. Fifteen rats were anaesthetized with 10 mg/kg body weight of xylazine hydrochloride and 50 mg/kg ketamine hydrochloride. Their dorsum was prepared for aseptic surgery. Then, paravertebral (5 cm) full thickness skin incisions were made on the animals using sterile scalpel blade. The incisions were sutured using size 2/0 silk thread with sterile swaged-on traumatic needle. Three treatment groups consisting of 5 animals per group were treated as follows: groups A and B were dosed orally with 300 mg/kg of 20% and 10% of the extract, respectively, while group C was given 1 ml/kg of sterile normal saline. The animals were treated daily for a period of 7 days. Sutures were removed at 8 dpw, and the wound breaking strength determined at 10 dpw using the constant water flow technique [35].

#### Dead space wound

Dead space wound were created following the method described by Rathi *et al*. [34]. Fifteen rats were randomly assigned into 3 groups of 5 animals per group. Under general anaesthesia (achieved using 10 mg/kg body weight of xylazine hydrochloride and 50 mg/kg ketamine hydrochloride), subcutaneous dead space wound were created in the region of the axilla by making a pouch through a small nip in the skin. Granuloma formation was induced by implanting sterile cotton pellets (30 mg) one on each axillae. The wounds were sutured and mopped with alcoholic swab. The animals were placed individually in a metal cage after grouping them to avoid them biting each other’s wound. Groups D and E were administered orally with 300 mg/kg of 20% and 10% of the extract, respectively, while group F was given 1 ml/kg of sterile normal saline, for 8 days. At day 10, rats were euthanatized and the cotton pellets together with the granuloma tissues were carefully removed, dried in the oven at 60°C, weighed and compared with the control.

#### Statistical analysis

The data obtained were analysed using one way analysis of variance (ANOVA) and a value of p < 0.05 was considered significant.

## Results

### Extraction

The methanolic leaf extract of *P. lappacea* had an aromatic smell and was greenish-black in colour. The percentage yield was 13.5% *w/w* material.

### Acute toxicity test

Administration of methanolic leaf extract of *P. lappacea* suspended in DMSO to rats even at the highest dose of 2000 mg/kg did not produce any death in the treated groups. No sign of acute toxicity was also observed.

### Phytochemical analysis

Preliminary phytochemical analysis qualitatively revealed the presence of steroids, glycosides, saponins, flavonoids, alkaloids, sugar and phenol. These were ascertained from the reactions involving colour changes, haemolysis, turbidity, layers, emulsification and precipitation (Table 1).

**Table 1:** **Phytochemical analysis of *P*. *lappacea *** **extract**

**Phytoconstituent**	**Amount**
Steroids	+++
Glycosides	+++
Saponins	++
Alkaloids	+++
Sugar	++
Phenol	+
Tannins	-

Keys: *+++* = appreciable amount, *++* = moderate amount, *+* = trace amount, - = completely absent.

### Minimum inhibitory and bactericidal concentrations of ***P. lappacea*** extract on wound isolates

The MIC values obtained for the *P. lappacea* extract against *P. aeruginosa*, *S. aureus* and *B. subtilis* was 9 mg/ml, 4 mg/ml and 3 mg/ml, respectively, while the corresponding MBC were 10 mg/ml, 8 mg/ml and 7 mg/ml, respectively (Table 2).

**Table 2:** **Minimum inhibitory and bactericidal concentrations of *P*. *lappacea *** **extract on tested bacterial organisms**

**Bacterial organism**	**MIC (mg/ml)**	**MBC (mg/ml)**
*Pseudomonas aeruginosa*	9	10
*Staphylococcus aureus*	4	8
*Bacillus subtilis*	3	7

Key: *mg/ml*, milligram per millilitre.

### Wound contraction

The percentage wound contraction in group I (20% extract treated) at 7 dpw was significantly (p < 0.05) high compared to groups II (10% extract treated), III (gentamicin treated) and IV (soft white paraffin treated). At 16, 19 and 21 dpw, though the percentage wound contraction in group I was the highest, it was not statistically significant (p > 0.05) compared to that of group III, but the percentage wound contraction of animals in groups I and III were significantly (p < 0.05) higher than those in groups II and IV. Animals in group IV showed significantly (p < 0.05) the least contraction rate throughout the study period. For all groups, most wound contraction occurred between 7 and 21 dpw (Table 3).

**Table 3:** Percentage wound contraction in rats post infliction of excision wounds

**Group (Treatment)**	**Mean ± standard error of mean wound contraction at days post wounding**							
	**7**	**10**	**13**	**16**	**19**	**21**	**24**	**27**
I (20% PLLEO)	18.0 ± 3.5^a^	35.6 ± 5.3	35.7 ± 6. 8	39.9 ± 0.7^a^	70.2 ± 13.4^a^	79.9 ± 13.4^a^	100 ± 0.0^a^	100.0 ± 0.0^a^
II (10% PLLEO)	8.1 ± 1.2^b^	39.8 ± 2.2	22.6 ± 2.8	22.9 ± 4.3^b^	35.7 ± 5.8^b^	44.2 ± 7.4^b^	89.0 ± 1.0^a^	100.0 ± 0.0^a^
III (Gentamicin ointment)	7.9 ± 1.5^bc^	36.7 ± 6.8	26.8 ± 5.4	36.7 ± 8.0^a^	63.3 ± 15.4^a^	70.9 ± 13.0^a^	100.0 ± 0.0^a^	100.0 ± 0.0^a^
IV (Normal saline)	8.7 ± 3.0^bd^	25.4 ± 4.6	23.3 ± 5.4	21.0 ± 4.9^c^	22.4 ± 5.7^c^	47.8 ± 14.8^b^	65.1 ± 10.4^b^	87.0 ± 8.3^b^

Key: *PLLEO* = *Pupalia lappacea* leaf extract ointment.

Different superscripts ^a,b,c,d^ across a column indicate significant difference in means at p < 0.05.

### Wound epithelialization

Epithelialization occurred between 18 and 25 dpw (Table 4). Epithelialization was significantly (p < 0.05) faster in animals in group I compared with those in groups II and IV. There was no significant difference (p > 0.05) in epithelialization time of wound between animals in groups I and III.

**Table 4:** Epithelialization time of rats post infliction of excision wound

**Group (Treatment)**	**Mean ± standard error of mean epithelialization time (days)**
I (20% PLLEO)	18.6 ± 0.5^a^
II (10% PLLEO)	22.0 ± 0.1^b^
III (Gentamicin ointment)	19.6 ± 0.3^a^
IV (Normal saline)	25.2 ± 0.2^b^

Key: *PLLEO* = *Pupalia lappacea* leaf extract ointment.

Different superscripts ^a,b^ across a column indicate significant difference in means at p < 0.05.

### Wound bacterial load

At 4 dpt, mean total viable count obtained in the treated groups (I, II and III) were significantly (p < 0.05) lower compared against the untreated control. Similar trend was observed at 7 and 14 dpt (Table 5).

**Table 5:** Total viable bacterial cell population from excision wounds of rats

**Group (Treatment)**	**Mean ± standard error of mean total viable count at days post-treatment**		
	**4**	**7**	**14**
I (20% PLLEO)	5.00 ± 79^a^	4.50 ± 87^a^	0.30 ± 20^a^
II (10% PLLEO)	6.00 ± 1.40^a^	3.80 ± 0.41^a^	0.70 ± 0.25^a^
III (Gentamicin ointment)	6.30 ± 1.46^a^	4.20 ± 2.28^a^	0.80 ± 0.44^a^
IV (Normal saline)	17.90 ± 2.76^b^	6.10 ± 1.62^b^	1.40 ± 0.33^b^

Key: *PLLEO* = *Pupalia lappacea* leaf extract ointment.

Different superscript ^a,b^ across a column indicate significant difference in means at p < 0.05.

### Incision wound

The wound breaking strength results showed a significant (p < 0.05) increase in the healed wounded tissue in groups A and B (extract treated) when compared against the control (Table 6).

**Table 6:** Wound breaking strength post infliction of incision wound

**Group (Treatment)**	**Mean ± standard error of mean wound breaking strength**
A (300 mg/kg of 20% PLLE)	813.8 ± 57.2^a^
B (300 mg/kg of 10% PLLE)	754.3 ± 62.5^a^
C (Control)	478.8 ± 19.1^b^

Key: *PPLE* = *P. lapaccea* leaf extract.

Different superscripts ^a,b^ across a column indicate significant difference in means at p < 0.05.

### Dead space wound

The result of the effect of the methanolic leaf extract of *P. lappacea* on granuloma weight showed that the weight of granuloma tissue of animals in group D (300 mg/kg of 20% extract treated) was significantly (p < 0.05) higher than those in groups E and F (control) (Table 7).

**Table 7:** Mean granuloma weight post infliction of dead space wounds

**Group (Treatment)**	**Mean ± standard error of mean granuloma weight**
D (300 mg/kg of 20% PLLE)	74.0 ± 4.3^b^
E (300 mg/kg of 10% PLLE)	70.8 ± 1.8^a^
F (Control)	70.8 ± 1.8^a^

Key: *PLLE* = *P. lapaccea* leaf extract.

Different superscript ^a,b^ across a column indicate significant difference in means at p < 0.05.

## Discussion

Wound healing according to Al-Henhena *et al*. [36] is a complex and dynamic process by which cellular structures and tissue layers in a damaged tissue restores itself as closely as possible to its original state. Wound contraction begins a week post wounding at the fibroblastic stage with the wound area undergoing shrinkage [36,37]. In the present study, topical application of *P. lappaceae* extract significantly accelerated wound healing with 20% ointment having the highest percentage wound contraction ability followed by gentamicin (a commercially sold antimicrobial agent used in dressing wounds) and 10% ointment of *P. lapeacae*. Wound contraction is made possible either due to an enhanced activity of fibroblast in regenerated wound tissue [38]. Myofibroblasts are believed to play a key role in wound contraction by exerting tension on surrounding extracellular matrix and secreting collagen which stabilizes the contraction [39]. The contracture potency of wounds might also be due to the ability of the wound dressing agents to enhance the synthesis in addition to collagen, fibronectin transforming growth factor BI [40]. There is also a report on increase levels of hexosamine and uric acid (matrix molecules) during early stage of wound healing [41]. In view of the above reports, the wound contraction and healing effects of *P. lappacea* might be attributed to its ability to stimulate and increase the synthesis of one or more of the above mentioned cells and substances/factors known to promote wound healing.

Stimulation of epithelial cell proliferations and angiogenesis are vital for wound healing process to take place [42]. Wound epithelialization is a process whereby there is epithelial regeneration post wounding with the epithelial cells proliferating and migrating over the wound bed, thereby providing a protective cover for the freshly formed tissues [43]. *P. lapaceae* extract might have accelerated epithelial regeneration due to its ability to promote angiogenesis and collagen formation and depositions [44,45]. According to Cohen *et al*. [46], collagen is a principal component of connective tissue and provides a structural framework for the regenerating tissue. Angiogenesis in granulation tissues on the other hand improves circulation to the wound site thereby providing oxygen and nutrients necessary for healing process that include re-epithelialization [47].

Wound tensile strength which is one of the indices for assessing wound healing is determined by the amount of fibroblast proliferation, collagen synthesis and neovascularization [38]. In this study, two models viz incisional and dead space wound were used to assess the effect of the methanolic leaf extract of *Pupalia lappaceae* on the tensile strength of the healed tissues. According to Kumar *et al*. [48], collagen which principally strengthens the wound and support extracellular tissue is composed of amino acids. Nayak *et al*. [32] also documented that the increase in the granuloma weight is associated with the presence of high content of protein. The pro-healing property of the extract by these facts might be attributed to its ability to promote protein synthesis leading to increase in the tensile strength. This ability to promote protein synthesis was dose dependent since the group that received 20% extract did better than the group that was given 10% of the extract. Although the mechanism of the increase in the tensile strength was not investigated in this study, the increased tensile strength could further be attributed to the ability of the extract to increase the Van der waals force interaction among the hydrogen ion bonds of the tripled helix collagen, leading to twisting of the collagen fibers [47]. The more the twisting of these collagen fibers, the greater the tensile strength and invariably the better healing of wounds [47].

The significant decreases in wound bacterial load in the treated groups suggest that the extract has *in vivo* antibacterial effect against the tested microorganisms which are involved in wound contamination. The extract was able to reduce wound bacterial load comparable to the reference drug, gentamicin. The antibacterial activity of the extract is further supported by the MIC result which showed that *P. lappacea* leaf methanolic extract exhibited bacteriostatic activity against the organisms at the concentrations shown in Table 2. The corresponding MBCs of 10 mg, 8 mg/ml and 7 mg/ml, respectively, further suggest that the extract was bacteriostatic at lower concentrations but bactericidal at higher concentrations.

The understanding and control of microbial infection of wounds is very important for better healing and its management [49]. Post operative wounds are usually infected by bacterial organisms [50]. Signs of bacterial infection of wounds are restlessness and scratching/biting of wound site [36]. Other signs include delay in the onset of the proliferative and remodeling phases of wound healing process due to the release of free radical and lytic enzymes at the wound site [51]. The delay in the wound healing by these free radicals is achieved by the ability of the free radical to destroy the lipids, proteins and extracellular matrix [52]. The absence of irritation and/or pain at wound site during treatment and the significant increase in the rate of wound contraction and wound re-epithelialization is a reflection of good antibacterial potentials of the plant as contained in the *in vivo* and *in vitro* antibacterial assay results. The antibacterial activity exhibited by the *P. lappacea* leaf extract could be attributed to its good antioxidant property and pytochemical constituents [53]. In this study, phytochemical analysis showed that *P. lappacea* methanolic leaf extract contained flavonoids, and flavonoids isolated from medicinal plants have been shown to possess antimicrobial activities [54]. Antioxidants have been reported to play a significant role in wound healing process by protecting tissues from oxidative damage [55]. It has been documented that plants with antibacterial and antioxidant properties enhance wound healing by accelerating wound contraction and re-epithelialization [31,52,56].

## Conclusion

The methanolic leaf extract of *Pupalia lappacea* showed remarkable dose dependent effect on wound contraction, wound epithelialization and wound tensile strength. The antibacterial studies showed that the plant exhibited good *in vivo* and *in vitro* antibacterial effects. These results validate the use of this plant in folkloric medicine for the treatment of wounds.

## Competing interests

The authors declare that they have no competing interests.

## Authors’ contributions

SOU: Conceived the study, designed, coordinated, carried out the extraction, reconstituted the extract, helped in wound creation and drafting of the manuscript. RIU: Anaesthetized the animals, participated in wound creation and helped in drafting the manuscript. JCM: Sourced the microorganisms, infected the animals, carried out the wound TVC, determined the MIC and MBC of the extract, and helped in drafting the manuscript. MUA: Helped in drafting the manuscript. CON: Carried out the phytochemical analysis of the extract. All authors read and approved the final manuscript.

## Pre-publication history

The pre-publication history for this paper can be accessed here:

http://www.biomedcentral.com/1472-6882/14/157/prepub
